# *Hibiscus sabdariffa* calyx aqueous extract mitigates alcohol withdrawal-induced anxiety and oxidative stress in mice

**DOI:** 10.1016/j.bbrep.2025.102423

**Published:** 2026-01-06

**Authors:** Nadège Emégam Kouémou, Louis Aimé Sepi, Mireille Sylviane Nguepi Dongmo, Ndzweng Linda Tamanji, Franklin Savo Mbeboh, Stephanie Jacqueline Kameni Ndjapdounke, Paul Aimé Noubissi, Bernard Tiencheu, Elisabeth Ngo Bum

**Affiliations:** a Department of Animal Biology and Conservation, Faculty of Science, University of Buea, Cameroon P.O. Box 63, Cameroon; b Department of Biological Sciences, Faculty of Science, University of Ngaoundéré, P.O. Box 454, Ngaoundéré, Cameroon; c Department of Biochemistry and Molecular Biology, Faculty of Science, University of Buea, P.O. Box 63, Cameroon; d Department of Biological Sciences, Faculty of Science, University of Maroua, P.O. Box 52, Maroua, Cameroon

**Keywords:** Alcohol withdrawal, Anxiety, Brain, *Hibiscus sabdariffa*, Liver

## Abstract

**Background:**

Alcohol withdrawal syndrome (AWS) happens following a sudden interruption of chronic alcohol intake. AWS is a severe condition, often leading to anxiety and seizures. Current treatments against AWS do not target all the features of the disease. This study aimed to evaluate *Hibiscus sabdariffa* aqueous extract on AWS in mice.

**Methods:**

Thirty-five male mice were grouped into seven sets of 5 animals. Each set (except the sham control) received alcohol (5 %) as drinking water and, in addition, alcohol at increasing concentrations (5 %–35 %, 0.4 g/kg to 2.8 g/kg) once every 24 h for 28 days. After alcohol weaning on day 29, anxiety was evaluated (days 29–31). Following behavioural recording, animals were euthanised. Brain and liver homogenates were used for biochemical evaluation of oxidative stress parameters. Alanine aminotransferase and Aspartate aminotransferase were assessed in the serum.

**Results:**

Alcohol withdrawal led to a significant (P < 0.001) decrease in open arm activities in the elevated plus maze. *Hibiscus sabdariffa* administration reversed the ethanol effect and increased open arms stay and exploration. *Hibiscus sabdariffa* also significantly (P < 0.05) increased center exploration of the open field, which was reduced by alcohol withdrawal. A treatment with *Hibiscus sabdariffa* significantly (P < 0.01) reduced the increase of brain and liver oxidation induced by alcohol withdrawal. Serum Alanine aminotransferase level was also significantly (P < 0.001) decreased by *Hibiscus sabdariffa* extract.

**Conclusion:**

This study's results justify the traditional utilisation of the drinks prepared from *Hibiscus sabdariffa* cayxes in treating patients suffering from AWS.

## Highlights

• *Hibiscus sabdariffa* calyx aqueous extract reverses alcohol withdrawal anxiety.
• *Hibiscus sabdariffa* administration protects the brain against ethanol-induced oxidative stress.
• *Hibiscus sabdariffa* calyxes' treatment protects the liver against ethanol toxicity.
• The protective effect of *Hibiscus sabdariffa* against ethanol-induced organ damage is due to its richness in antioxidant phytomolecules.

## Graphical abstract

**Figure:**
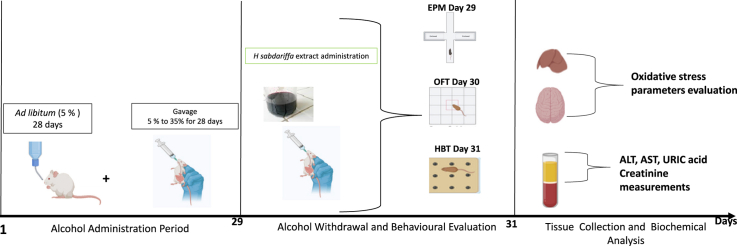

## 1 Introduction

Alcoholism is a serious health and social concern worldwide [1,2]. Alcohol addiction causes the death of over 3 million people each year [3]. In Sub-Saharan Africa, the burden of alcohol use disorders is alarming [4]. If nothing is done, the consequences of this alcohol dependence will increase the number of psychiatric disorders among African populations in the years to come [5]. One common characteristic of alcohol dependence is the persistence of the drinking behaviour despite the psychological, behavioural, and social consequences [6]. The first attempt to slow down the consequences of chronic alcoholism is through the reduction of the amount of alcohol intake in each patient [7]. This reduction of chronic alcohol consumption in alcohol-dependent individuals without proper medical management frequently leads to alcohol withdrawal syndrome (AWS) [7,8]. AWS is a life-threatening situation that occurs in about 25 per cent of hospitalised alcohol abusers [9]. AWS manifestations include seizures, hypertension, vomiting, and, most often, anxiety [10,11]. Alcohol affects several neurotransmitter systems in different brain regions. GABA, glutamatergic, dopaminergic, and serotonergic neurotransmission are altered by alcohol intake [10]. Regular alcohol consumption leads to the desensitisation of GABA A receptors, while the NMDA receptors of glutamate are upregulated. During alcohol withdrawal, the sudden termination of alcohol breaks this long-term neuroadaptation [12]. The prominent effect observed includes an exacerbation of the general excitation of the brain [9,13]. This hyperglutamatergic function has been associated with neuropsychiatric conditions and autonomic symptoms occurring during AWS [13,14]. The molecular basis for explaining this disruption in homeostasis includes an increase in the brain's inflammation and oxidative stress states [15,16].

The N-methyl-d-aspartate antagonist blocker acamprosate and benzodiazepines (lorazepam and diazepam) are the medications usually prescribed for acute and severe AWS [8,17]. Benzodiazepines are associated with over-sedation, high risk of developing addiction, and chronic liver disease [18].

The liver is responsible for alcohol breakdown. It contains the enzyme alcohol dehydrogenase, which metabolises alcohol [19]. Acetaldehyde, the end product of this enzymatic action, is very toxic to liver cells [20,21]. Chronic alcohol consumption is one of the leading causes of hepatic failure [22]. It has been exhaustively outlined that oxidative stress is one of the fundamental pathways by which alcohol leads to both liver and brain toxicity [21,23]. These discoveries have guided an intensive screening of new pharmacological compounds with antioxidant properties to mitigate alcohol-induced multi-organ damage [24].

*Hibiscus sabdariffa* (*H. sabdariffa*) is an edible green vegetable of the family Malvaceae. *H. sabdariffa* is also named “Hibiscus,” “Sour tea,” or “Roselle.” Different parts of Roselle are used as food, for preparing soft and refreshing drinks, and for medicine [25]. *H. sabdariffa* is the principal ingredient of the “Zobo” drink, a popular refreshing tea in Nigeria [26]. The calyxes of Roselle are the main constituent of “Bissap,” a famous natural drink in West and Central Africa consumed for its richness in nutrients, refreshing, and medical properties [27]. Literature reports the uses of the extract prepared from the calyxes of Roselle as a natural cure against metabolic diseases, including hypertension and diabetes mellitus [28,29]. Hibiscus calyxes have been shown to have antibacterial properties against *Escherichia coli* [30]. As phytochemicals, *H sabdariffa* contains flavonoids, alkaloids, hydrobenzoic acid, and anthocyanins [26,31]. Numerous studies have shown that Roselle is effective against degenerative diseases due to oxidative stress [31]. In Cameroon, a tea called “Foléré” prepared from the calyxes of *H. sabdarifa* is used by the population of the Nord region to relieve patients suffering from alcohol intoxication. Despite this common empirical knowledge about the uses of the drink prepared with *H. sabdarifa* calyxes to treat people suffering from alcohol damage in the Nord-Cameroon, the scientific basis validating this traditional medicine is lacking. Therefore, this study was initiated to assess the effect of *H. sabdariffa* calyx on alcohol withdrawal-induced anxiety and oxidative stress in mice.

## 2 Materials and methods

### 2.1 Plant

*H sabdariffa* calyxes were purchased in the Muea (South West Cameroon) market in July 2021. Dr Djibrilla Mana, a botanist at the University of Buea, identified the Species taxonomically. The plant was compared to the voucher specimen identified and kept at the Cameroon National Herbarium, Yaounde, under reference 25776/SFR/Cam. Calyxes of the plant were ground using an electric blender. Two hundred and 86 g (286 g) of the calyx powder were infused for 24 h into 2860 mL of boiled (100 °C) distilled water. The reddish filtrate was lyophilised. The total mass of the red-brown solid obtained was 20.5 g. The extraction yield was then 7.16 %.

### 2.2 Animals and ethical statements

The experimental animals were from the University of Buea (Cameroon) animal facility. Mice were two months old and weighed between 20 and 25 g at the start of the experiment. They were housed in polyethene cages with white sawdust as bedding. Animals were housed two per cage and had food and water *ad libitum*. Mice were acclimated for one week in the laboratory before the start of the experiment. The standard operating procedure followed the guidelines of the University of Buea Institutional Animal Care and Use Committee (UB-IACUC N֩8/2021). The National Institute of Health (NIH) protocol for using laboratory animals was followed.

### 2.3 Drugs and chemicals

Alcohol (Ethanol 95 %) and Hydrogen peroxide (3 %) were bought locally from Salvation Pharmacy (Buea, Cameroon). Diazepam was purchased from Roche Laboratories (France). Ellman reagent (DTNB), naphthyl ethylenediamine, phosphoric Acid, sulphanilamide, thiobarbituric Acid, Trizma base, and trichloroacetic Acid were all from Sigma (Germany). Total protein, Uric acid, Creatinine, ALT, and AST assay kits were purchased from Chronolab (Spain).

### 2.4 Animal grouping and experimental design

Swiss mice (35 males, age two months) were assigned to seven groups of five mice. A normal control group, a negative control group, four (4) test groups, and a positive control group (Table 1).

**Table 1:** Animal grouping and treatment regimen.

Group	Treatment
Normal control (DW + DW)	Distilled water (10 mL/kg) as treatment throughout the experiment
Negative control (ALC + DW)	Alcohol (0.4–2.4 g/kg) by gavage from day 1 to day 28 + distilled water (10 ml/kg) after alcohol withdrawal (days 29–31).
Test groups I, II, III, and IV (ALC + HS)	Alcohol (0.4–2.4 g/kg) by gavage from day 1 to day 28 + *H. sabdariffa* at different doses after alcohol withdrawal (days 29–31).
Positive control (ALC + DZP)	Alcohol (0.4–2.4 g/kg) by gavage from day 1–28 + diazepam by gavage after alcohol withdrawal (day 29–31).

ALC: Alcohol; DW: Distilled water; DZP: diazepam; HS: *Hibiscus sabdariffa*.

Mice in the normal control were gavaged with distilled water. From day 1 to day 7, mice in the negative control, test groups, and the positive control received 5 % (0.4 g/kg) alcohol by gavage once every 24 h. In addition, they had 5 % alcohol as drinking water *ad libitum* from days 1–28. From day 8 to day 14, mice in these same groups were treated with 10 % (0.8 g/kg) alcohol solution by gavage, 20 % (1.6 g/kg) alcohol solution from day 15 to day 21, and 35 % (2.8 g/kg) alcohol solution from day 22 to day 28. On day 29, alcohol was withdrawn in all groups. Following ethanol withdrawal, mice in the test groups received *H. sabdariffa* calyxes at 50, 100, 200, and 400 mg/kg doses. The positive control group mice received diazepam 3 mg/kg (EPM) and 0.3 mg/kg (OFT and HBT). Mice in the negative control group were given 10 mL/kg of distilled water as treatment. Behavioural tests were performed during the withdrawal time: the EPM (day 29), the OFT (day 30), and the HBT (day 31), 1 h after treatment.

### 2.5 Qualitative identification of phytochemicals

Qualitative colourimetric methods were used to identify alkaloids, tannins, saponins, flavonoids, and phenolic compounds in Hibiscus calyx aqueous extract.

#### 2.5.1 Alkaloids

This test was performed using Dragendoff's reagent. To 1 mL of *H. sabdariffa* extract, five drops of Dragendoff's reagent were added. Alkaloid presence was confirmed by the appearance of an orange-brown colouration [32].

#### 2.5.2 Phenolic compounds

Three droplets of ferric chloride (FeCl_3_) solution (5 %) were added to two mL of the calyxes extract solution. The solution was blue-black or green in the presence of polyphenolic compounds [33].

#### 2.5.3 Flavonoids

One mL of the calyx extract solution was added to a few droplets of 1/10 M sodium hydroxide. Flavonoids were highlighted by a yellow precipitate, which disappears when Shinoda's reagent was introduced into the medium [34].

#### 2.5.4 Saponins

Saponosides dissolve in water, forming a foaming solution. The aqueous extract was mixed with 2 mL of water and heated for 5 min. After shaking, the foam's persistence for 5 min indicates the presence of saponins in the medium [35].

#### 2.5.5 Tannins

Three droplets of 1 % ferric chloride (FeCl3) were added to one mL of the extract solution. Tannins in the medium were revealed by a blue-black or brownish-green colouration [35].

### 2.6 Behavioural assessments

#### 2.6.1 Elevated plus-maze (EPM)

The EPM was made up of white wood. The apparatus had two open arms (16 × 5 cm) and two closed arms (16 × 5 × 10 cm) joined by a central square (5 × 5 cm). Animals were introduced individually into the paradigm for 5 min of free exploration. The cumulative time of visits to the arms, as well as the number of arm entries, was noted. The maze was wiped using a 5 % acetic acid solution after each recording. The EPM test was performed on day 29, as previously described by Moto and co-workers in 2018 [36].

#### 2.6.2 Open-field test (OFT)

This test provides a qualitative and quantitative measure of exploratory and locomotor activity in rodents [37]. The OFT was a square enclosure made of wood with high edges. The floor of this enclosure has 17 squares: 16 squares that divide the inner surface of the apparatus and one (1) central square. The dimensions of the experimental apparatus used in our research were 40 × 40 × 45 cm. On day 30 of the assessment, the mice's behaviours were recorded in this paradigm for 5 min. Parameters recorded include line crossings (counts), grooming (counts), rearing (counts), centre time (seconds), and faecal boli(counts).

#### 2.6.3 Hole-board test (HBT)

Boissier and Simon, in 1962, first introduced HBT, which was further developed by File and Wardill in 1975 to evaluate anxiety in rodents [38]. The hole board was a plywood square (40 × 40 × 25 cm) with 16 pits (3 cm diameter each) equally distributed on the inner surface of the maze. The HB apparatus had a height of 25 cm from the floor level. The HBT was performed on day 31. Line crossings (counts), head dipping (counts), and the latency (seconds) to the first head dipping were recorded for 5 min.

### 2.7 Biochemical assessments

On day 32 of experimentation, mice were euthanised by cervical dislocation by a well-trained and experienced operator. Livers and brains were quickly removed and used to prepare a 20 % homogenate. Blood was centrifuged at 3000 revolutions/min for 30 min to obtain serum.

#### 2.7.1 Malondialdehyde (MDA) level determination

Lipid peroxidation in the targeted organs was measured using the thiobarbituric assay [39]. MDA content is expressed in μmol/g of tissue.

#### 2.7.2 Determination of Nitric Oxide (NO) contents

The Greiss reagent was used to determine nitric oxide in supernatants. The nitric level (in mol/of tissue) was determined using a sodium nitrate standard curve [40].

#### 2.7.3 Evaluation of Reduced Glutathione (GSH) level

GSH was assessed in samples using DTNB as initially described by Ellman [41]. GSH content in tissue was expressed as μmol/g of protein.

#### 2.7.4 Evaluation of Superoxide Dismutase (SOD) activity

SOD activity was determined based on Misra and Fridovich's protocol [42]. SOD activity is expressed as U/mg of tissue.

#### 2.7.5 Determination of catalase activity

The method described by Sinha and collaborators in 2014 was used to determine catalase activity in brain and liver homogenates [43]. Catalase activity was expressed in nmol/g of protein.

#### 2.7.6 Assessment of Alanine Aminotransferase (ALT) levels

ALT concentration was measured using Chronolab assay kits. 0.5 mL of working reagent and 50 μL of sample were introduced into a 1 cm cuvette. After homogenisation and incubation for 1 min. The absorbance was read at 350 nm during 1-min intervals for 3 min. ALT level was expressed as U/L of serum.

#### 2.7.7 Assessment of Aspartate Aminotransferase (AST) levels

AST concentration was determined using Chronolab assay kits. 0.5 mL of working reagent and 50 μL of sample were mixed. After homogenisation and incubation for 1 min. The absorbance was recorded at 350 nm during 1-min intervals for 3 min. AST level expressed as U/L of serum.

#### 2.7.8 Assessment of creatinine levels

Creatinine was assessed using Chronolab assay kits following the manufacturer's specifications. 0.5 mL of working reagent was mixed with 50 μL of serum. After the homogenisation of the solution, the absorbance was recorded at 492 nm after 30 s and 90 s. Creatinine level was expressed in mg/dL.

#### 2.7.9 Measurement of uric acid levels

Uric acid was determined using Chronolab assay kits following the manufacturer's specifications. The working reagent (0.1 mL) and 25 μL of the sample were mixed. After incubation for 5 min, the absorbance was read at 520 nm. Uric Acid content of the serum was expressed in mg/dL.

### 2.8 Statistical analysis

The data generated were analysed with GraphPad Prism 8.4.3(686). Data were tested for normality using the Shapiro-Wilk test and homogeneity of variance using the Brown-Forsythe test before ANOVA. ANOVA followed by Tukey's correction was used to compare means. Data depict the means (n = 5) ± standard error of all raw values of treatment groups. For P ≤ 0.05, differences among treatments were noted as significant.

## 3 Results

### 3.1 Phytochemical screening of *H. sabdariffa* calyx

*H. sabdariffa* calyx aqueous extract contains: tannins, alkaloids, saponins, polyphenols, and flavonoids (Table 2).

**Table 2:** Phytochemical composition of *H. sabdariffa* calyxes.

Bioactive compounds	Alkaloids	Polyphenols	Flavonoids	Tannins	Saponins
Presence/Absence	+	+	+	+	+

(+): Present.

### 3.2 Effect of *H. sabdariffa* calyxes on the number of entries and the time spent in the open arms of the elevated plus maze

A one-way ANOVA results showed that there was a significant treatment effect among groups concerning the number of entries [F (6, 28) = 21.48; P < 0.0001] and time spent [F (6, 28) = 27.07; P < 0.0001] in the open arms of the EPM.

**Fig. 1:**
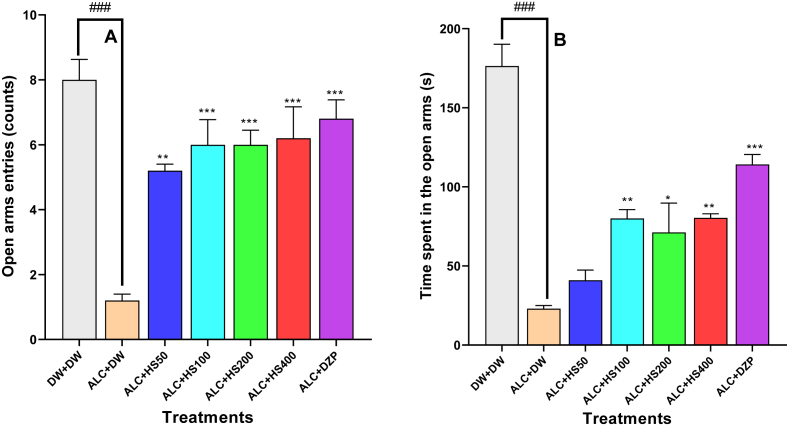
**Effect of *H. sabdariffa* calyxes on the number (A) and the time spent (B) in the open arm of the elevated plus maze** Each bar depicts the mean ± SEM. n = 5; ^###^P < 0.001vs DW + DW ∗P ≤ 0.05, ∗∗P ≤ 0.01, ∗∗∗P ≤ 0.001; vs ALC + DW. ALC: Alcohol; DZP: Diazepam; DW: distilled water; HS50: *Hibiscus sabdariffa;* 50 mg/kg, HS100: *Hibiscus sabdariffa* 100 mg/kg, HS200: *Hibiscus sabdariffa* 200 mg/kg, HS400: *Hibiscus sabdariffa* 400 mg/kg.

*H. sabdariffa* administration increased open arms entries, which were significantly reduced by ethanol administration. Open arms entries augmented from 1.20 ± 0.2 entries in the negative control to 6.00 ± 0.77, 6.0 ± 1.73, and 6.20 ± 0.97 entries in the *H. sabdariffa* group at doses of 100, 200, and 400 mg/kg, respectively. Diazepam increased open arms entries to 6.80 ± 0 58 (P ≤ 0.001) (Fig. 1A).

As seen in Fig. 1B, open arms time significantly increased from 23.00 ± 2.00 s in the negative control to 80.0 ± 5.65 s (P ≤ 0.01), 71 ± 18.5 s (P ≤ 0.01), and 80.4 ± 2.58 s (P ≤ 0.001) in *H. sabdariffa* groups at the doses of 100, 200, and 400 mg/kg, respectively. Diazepam increased open arm time to 114 ± 6.30 s (P ≤ 0.001).

### 3.3 Effect of *H. sabdariffa* calyx on lines crossing, number of rearing, number of grooming, time spent in the centre, and the faecal boli in the open field

Line crossing in the open field test was significantly affected by the various treatments [F (6, 28) = 14.70; P < 0.001]. Ethanol administration significantly reduced line crossing. The aqueous extract of *H. sabdariffa* calyces significantly (P ≤ 0.001) increased the line crossings compared to the negative control. Line crossings numbers were 117.00 ± 2.9 and 123.00 ± 9.2 in the groups treated with *H. sabdariffa* calyx extract at 200 or 400 mg/kg (P ≤ 0.001), respectively, compared to 66.60 ± 6.35 in the negative control. Diazepam significantly (P ≤ 0.001) increased the number of lines crossing to 129.00 ± 7.3 (Table 2).

The number of rearing was affected by group treatments [F (6, 28) = 16.70; P < 0.0001]. Rearing significantly (P ≤ 0.001) decreased from 12.80 ± 0.86 in the negative control group to 7.40 ± 0.86, 5.0 ± 0.63, or 5.80 ± 0.86 in *H. sabdariffa* extract-treated groups at 100, 200, or 400 mg/kg, respectively. Diazepam significantly (P ≤ 0.001) decreased rearing to 3.00 ± 0.3 (Table 2).

A one-way ANOVA results showed that there was a significant treatment effect among groups on grooming behaviour [F (6, 28) = 10.21; P < 0.0001]. Tukey's post hoc test showed that eTHANOL administration significantly decreased the number of grooming in mice, 0.8 ± 0.3 in the negative control, against 4.40 ± 0.60 in normal control mice. The extract of *H*. *sabdariffa* at 400 mg/kg, or diazepam, significantly increased grooming to 2.80 ± 0.49 (P ≤ 0.05) or 3.60 ± 0.40 (P ≤ 0.01), respectively (Table 2).

Negative control mice spent less time in the centre of the OFT when compared to the other groups [F (6, 28) = 6.081; P = 0.0004] (Table 3). *H. sabdariffa* at 400 mg/kg or diazepam (0.3 mg/kg) significantly increased the time spent in the centre of the OFT, respectively to 11.20 ± 1.53 (P ≤ 0.05) or 12.66 ± 0.97 (P ≤ 0.01) compared to 5.60 ± 0.92 s in the negative control mice (Table 3).

**Table 3:** Effect of *H. sabdariffa* calyxes on some recorded parameters of the Open field test.

Treatments	Lines crossing (counts)	Rearing (counts)	Grooming (counts)	Centre time (seconds)	Faecal boli (counts)	Total Peripheral time (seconds)
Normal control	145.00 ± 11	4.2 0 ± 0.37	4.40 ± 0.60	13.40 ± 1.54	0.6 ± 0.20	286.60 ± 1.54
Negative control	66.60 ± 6.35^###^	12.80 ± 0.89^###^	0.8 ± 0.30^###^	5.60 ± 0.92^###^	3.20 ± 0.86^#^	294.40 ± 0.37^###^
HS50	78.00 ± 4.64	9.20 ± 1.36	1.40 ± 0.24	8.80 ± 0.37	2.20 ± 0.86	291.20 ± 0.37^#^
HS100	97.20 ± 8.21	7.40 ± 0.87^∗^	1.00 ± 0.44	10.00 ± 0.83	0.40 ± 0.40^∗^	290.00 ± 0.83^∗^
HS200	177.00 ± 8.21^∗^∗∗	5.00 ± 0.63^∗^∗∗	1.60 ± 0.40	10.20 ± 0.53	1.60 ± 0.24^∗^	289.80 ± 0.51^∗^∗
HS400	123.00 ± 6.16^∗^∗∗	5.80 ± 0.86^∗^∗∗	2.80 ± 0.49^∗^	11.20 ± 1.53^∗^	0.40 ± 0.42^∗^	288.80 ± 1.33^∗^∗
DZP	129.00 ± 7.30^∗^∗∗	3.00 ± 0.30^∗^∗	3.60 ± 0.0^∗^∗	12.60 ± 0.98^∗^∗	0.60 ± 0.20^∗^	287.40 ± 0.98^∗^∗∗

Values are the mean ± SEM. n = 5; ^#^P < 0.05, ^###^P < 0.001vs Normal control ∗P ≤ 0.05, ∗∗P ≤ 0.01, ∗∗∗P ≤ 0.001; vs Negative control. DZP: Diazepam; HS50: *Hibiscus sabdariffa* 50 mg/kg; HS100: *Hibiscus sabdariffa* 100 mg/kg; HS200: *Hibiscus sabdariffa* 200 mg/kg; HS400: *Hibiscus sabdariffa* 400 mg/kg.

### 3.4 Effect of *H. sabdariffa* calyxes on the number of line crossings, the latency to the first head dipping, and the number of head dipping in the hole board test

**Fig. 2:**
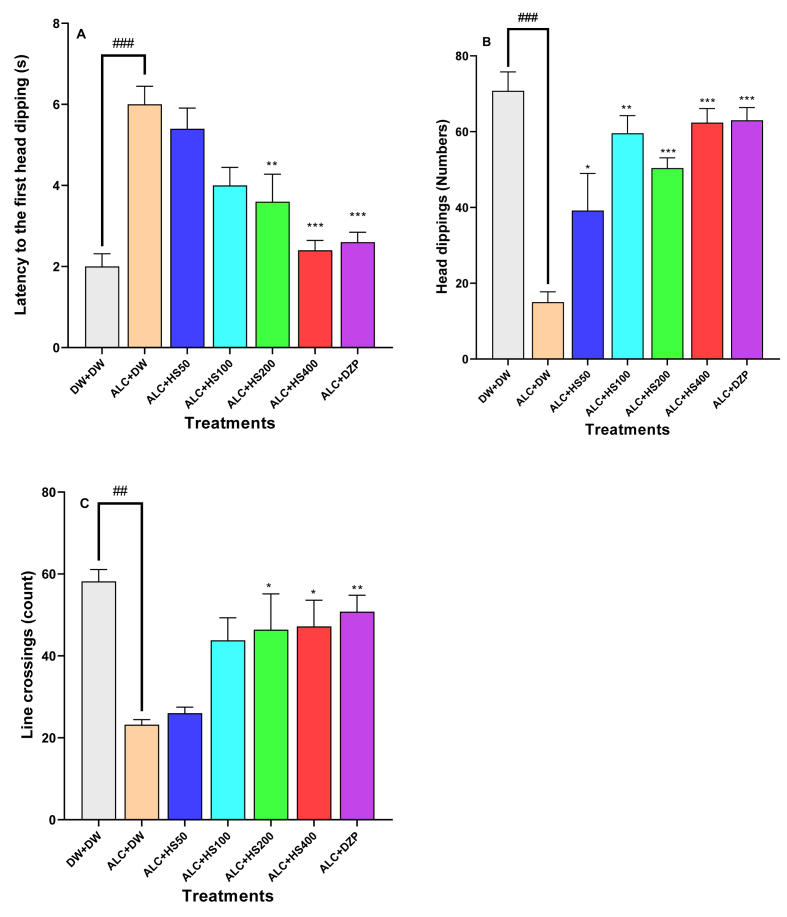
**Effect of *H. sabdariffa* calyxes on the number of line crossings, the latency to the first head dipping, and the number of head dipping in the hole board test** Each bar depicts the mean ± SEM. n = 5; ^###^P < 0.001vs DW + DW ∗P ≤ 0.05, ∗∗P ≤ 0.01, ∗∗∗P ≤ 0.001; vs ALC + DW. ALC: Alcohol; DZP: Diazepam; DW: distilled water; HS50: *Hibiscus sabdariffa*; 50 mg/kg, HS100: *Hibiscus sabdariffa* 100 mg/kg; HS200: *Hibiscus sabdariffa* 200 mg/kg, HS400: *Hibiscus sabdariffa* 400 mg/kg.

The results from the One-way ANOVA showed that there was a significant difference in the latency to the first head dipping among the treatment groups [F (6, 28) = 12.25; P < 0.0001]. ETHANOL withdrawal significantly increased the latency to the first head dipping in negative control mice. *H. sabdariffa* calyxes extract at 200 or 400 mg/kg significantly decreased this latency, respectively to 3.60 ± 0.67 s (P ≤ 0.01) or 2.40 ± 0.24 s (P ≤ 0.001), against 6.00 ± 0.44 s in the negative control mice (Fig. 2A).

Ethanol withdrawal significantly decreased the number of head dips in negative control mice. *H. sabdariffa* calyxes extract at 100, 200, or 400 mg/kg significantly increased it, respectively, to 59.6 ± 10.4 s (P ≤ 0.01), 50.4 ± 2.71(P ≤ 0.001) seconds, or 162.50 ± 3.72 s (P ≤ 0.001) compared to 15.00 ± 2.76 s in the negative control mice (Fig. 2B).

Ethanol withdrawal decreased the number of lines crossed in the negative control group when compared to the other groups [F (6, 28) = 14.7; P < 0.001]. Treatment with the aqueous extract of *H. sabdariffa* calyxes at 200 or 400 mg/kg significantly (P ≤ 0.001) antagonised the effect of ETHANOL by increasing the number of lines crossed to 46.40 ± 8.74 or 47.20 ± 6.59 s, as compared to 23.20 ± 1.24 s in the negative control (Fig. 2C).

### 3.5 Effect of *H. sabdariffa* calyxes on cerebral and hepatic markers of oxidative stress in mice subject to alcohol withdrawal

Statistical analysis with ANOVA demonstrated a significant effect of the various treatments on brain [F (6, 28) = 41.54; P < 0.0001] and liver [F (6, 28) = 93.75; P < 0.0001] MDA.

**Fig. 3:**
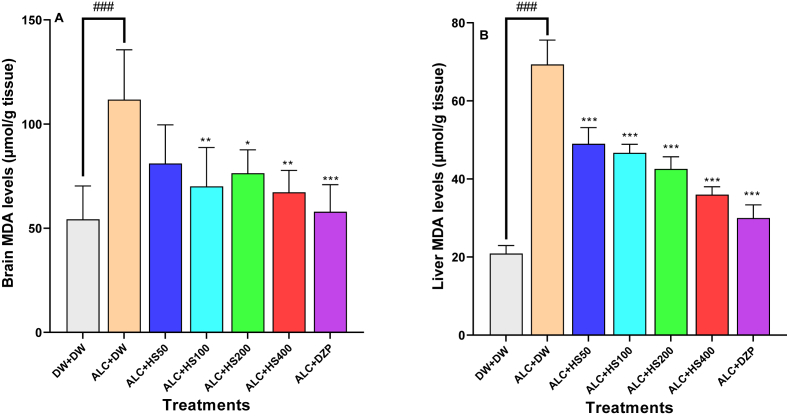
**Effect of *H. sabdariffa* calyxes on cerebral (A) and hepatic (B) levels of malondialdehyde** Each bar depicts the mean ± SEM. n = 5; ^###^P < 0.001vs DW + DW ∗P ≤ 0.05, ∗∗P ≤ 0.01, ∗∗∗P ≤ 0.001; vs ALC + DW. ALC: Alcohol; DZP: Diazepam; DW: distilled water; HS50: *Hibiscus sabdariffa;* 50 mg/kg, HS100: *Hibiscus sabdariffa*; 100 mg/kg, HS200: *Hibiscus sabdariffa* 200 mg/kg, HS400: *Hibiscus sabdariffa* 400 mg/kg.

Mice subjected to ETHANOL withdrawal presented a significant increase in the cerebral MDA: 112.00 ± 10.7 μmol/g in the negative control against 54.30 ± 7.17 μmol/g in normal control mice (Fig. 3A). *H. sabdariffa* calyxes aqueous extract at 100, 200 or 400 mg/kg significantly decreased the cerebral level of MDA to 81.0 ± 8.30 μmol/g, 70.01 ± 8.30 μmol/g, or 67.2 ± 4.69 μmol/g compared to 112.00 ± 10.7 μmol/g in the negative control (Fig. 3A). Diazepam at 0.3 mg/kg significantly (P ≤ 0.001) reduced the brain MDA level to 57.9 ± 5.81 μmol/g.

In the liver, MDA significantly (P ≤ 0.001) decreased from 69.40 ± 2.79 μmol/g in the negative control to 46.7 ± 0.96 μmol/g, 42.60 ± 1.40 μmol/g, or 36.0 ± 0.89 μmol/g in extract-treated groups at 100, 200, or 400 mg/kg, respectively. Diazepam administration significantly (P ≤ 0.001) reduced the liver MDA level to 30.0 ± 1.50 μmol/g (Fig. 3B).

### 3.6 Effect of *H. sabdariffa* calyxes on cerebral and hepatic levels of nitric oxide

One–way ANOVA showed that the brain [F (6, 28) = 415.4; P < 0.0001], the liver [F (6, 28) = 212; P < 0.0001] and nitric oxide content were affected by the various treatments.

**Fig. 4:**
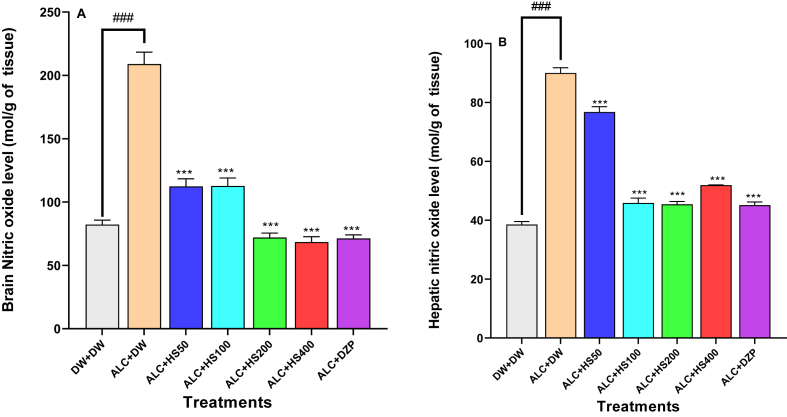
**Effect of *H. sabdariffa* calyxes on cerebral (A) and hepatic (B) levels of Nitric oxide** Each bar represents the mean ± SEM. n = 5; ^###^P < 0.001, compare to DW + DW ∗∗∗P ≤ 0.001; compare to the negative control (ALC + DW). ALC: Alcohol; DZP: Diazepam; DW: distilled water; HS50: *Hibiscus sabdariffa* 50 mg/kg; HS100: *Hibiscus sabdariffa* 100 mg/kg; HS200: *Hibiscus sabdariffa* 200 mg/kg; HS400: *Hibiscus sabdariffa* 400 mg/kg.

The brain, NO level decreased significantly from 209.06 ± 18.11 mol/g in the negative control group to 112.4 ± 24 mol/g (P ≤ 0.01), 112.7 ± 0.55 mol/g (P ≤ 0.05), 72.81 ± 1.64 mol/g (P ≤ 0.001) or to 68.41 ± 3.45 mol/g (P ≤ 0.001) in extract treated groups at 50, 100, 200 or 400 mg/kg, respectively (Fig. 4A).

After ETHANOL withdrawal, aqueous extract of calyxes of *H sabdariffa* at 100 or 200 mg/kg significantly decreased the hepatic NO levels, respectively, to 45.87 ± 3.68 or 45.39 ± 0.85 mol/g as compared to 76.74 ± 6.72 mol/g in the negative control group (Fig. 4B).

Diazepam at 0.3 mg/kg significantly (P ≤ 0.001) reduced hepatic and cerebral NO levels to 35.13 ± 5.39 mol/g (P ≤ 0.01) and 71.34 ± 10.27 mol/g, respectively (Fig. 4A and B).

### 3.7 Effect of *H. Sabdarifa* calyxes on brain and hepatic concentration of reduced glutathione

The statistical analysis results showed that both the brain [F (6, 28) = 27.7; P < 0.0001] and the liver [F (6, 28) = 37.4; P < 0.0001] content of reduced glutathione were affected by the various treatments.

**Fig. 5:**
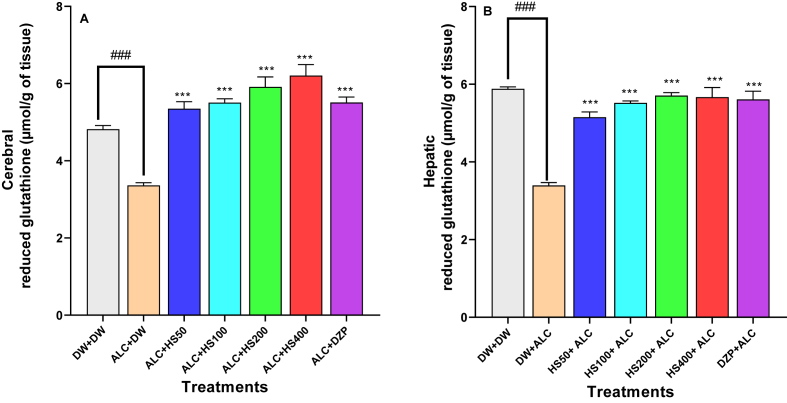
**Effect of *H. sabdariffa* calyxes on brain (A) and hepatic (B) concentration of reduced glutathione** Each bar represents the mean ± SEM. n = 5; ^###^P < 0.001, compare to DW + DW; ∗∗∗P ≤ 0.001; compare to the negative control (ALC + DW). ALC: Alcohol; DZP: Diazepam; DW: distilled water; HS50: *Hibiscus sabdariffa* 50 mg/kg; HS100: *Hibiscus sabdariffa* 100 mg/kg; HS200: *Hibiscus sabdariffa* 200 mg/kg; HS400: *Hibiscus sabdariffa* 400 mg/kg.

Ethanol withdrawal significantly decreased the concentration of brain reduced glutathione from 3.36 ± 0.06 μmol/g in the negative control group to 4.81 ± 0.09 μmol/g in the normal control mice. *H. sabdariffa* calyxes at 100, 200, or 400 mg/kg significantly increased this concentration, respectively, to 5.51 ± 0.09 μmol/g, 5.92 ± 0.25 μmol/g, or 6.21 ± 0.28 μmol/g (P ≤ 0.001). Diazepam also increased brain reduced glutathione concentration to 5.51 ± 0.14 μmol/g (P ≤ 0.001) (Fig. 5A).

*H. sabdariffa* at 100, 200, or 400 mg/kg, respectively, significantly (P ≤ 0.001) increased hepatic concentration of reduced glutathione to 5.52 ± 0.05, 5.71 ± 0.07, or 5.67 ± 0.24 μmol/g/g compared to 3.29 ± 0.16 μmol/g in the negative control group (Fig. 5B).

Diazepam (0.3 mg/kg) also increased reduced glutathione concentration in the liver to 5.61 ± 0.21 μmol/g.

### 3.8 Effect of *H. sabdariffa* calyxes extract on the cerebral and hepatic concentration of superoxide dismutase

**Fig. 6:**
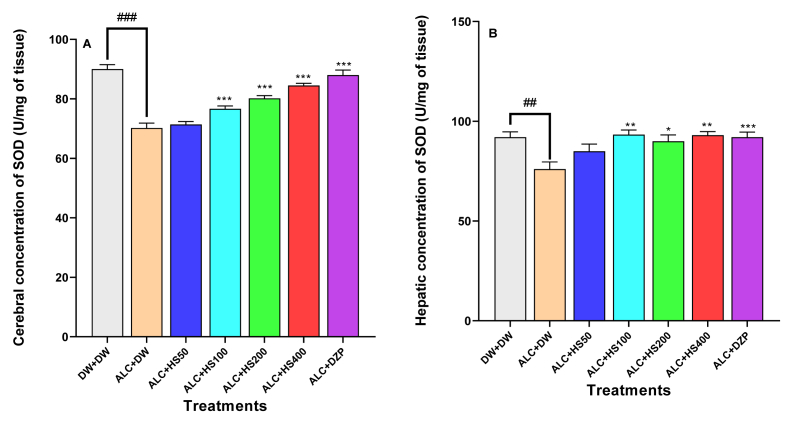
**Effect of *H. sabdariffa* calyx extract on the cerebral (A) and hepatic (B) concentration of superoxide dismutase** Each bar represents the mean ± SEM. n = 5; ^###^P < 0.001, compare to DW + DW ∗P ≤ 0.05, ∗∗P ≤ 0.01, ∗∗∗P ≤ 0.001; compare to the negative control (ALC + DW). ALC: Alcohol; DZP: Diazepam; DW: distilled water; HS50: *H sabdariffa* 50 mg/kg; HS100: *H sabdariffa* 100 mg/kg; HS200: *H sabdariffa* 100 mg/kg; HS400: *H sabdariffa* 400 mg/kg.

Ethanol administration decreased the brain concentration of SOD in negative control mice when compared to the other groups [F (6, 28) = 39.0; P < 0.0001] (Fig. 6A). *H. sabdariffa* aqueous calyxes extract significantly increased the brain SOD concentration to 76.70 ± 0.94 (P ≤ 0.05), 80.20 ± 0.89 (P ≤ 0.001), or 84.50 ± 0.71 U/mg (P ≤ 0.001), respectively, at 100, 200, or 400 mg/kg (Fig. 6A).

Hepatic SOD concentration decreased following ETHANOL administration [F (6, 28) = 4.83; P = 0.0017] (Fig. 6B). The plant extract at 100, 200, or 400 mg/kg significantly increased the hepatic SOD concentration, respectively to 93.33 ± 2.28, 90.15 ± 3.13, or 93.00 ± 1.79 U/mg compared to 76.00 ± 3.58 U/mg in the negative control group (Fig. 6B).

Diazepam significantly (P ≤ 0.01) increased the hepatic and cerebral SOD concentrations to 92 ± 2.57 and 88 ± 1.67 U/mg, respectively (Fig. 6A and B).

### 3.9 Effect of *H sabdariffa* calyx extract on the cerebral and hepatic activity of the catalase

**Fig. 7:**
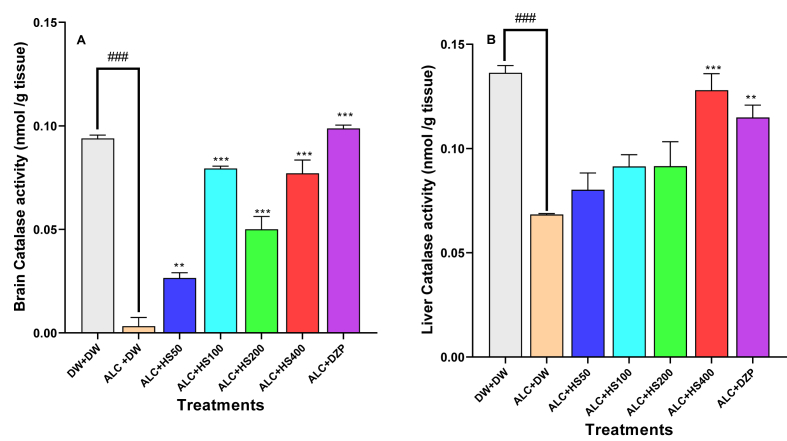
**Effect of *H. sabdariffa* calyxes extract on the cerebral (A) and hepatic (B) activity of catalase** Each bar represents the mean ± SEM. n = 5; ^###^P < 0.001, compare to DW + DW ∗∗P ≤ 0.01, ∗∗∗P ≤ 0.001; compare to the negative control (ALC + DW). ALC: Alcohol; DZP: Diazepam; DW: distilled water; HS50: *Hibiscus sabdariffa* 50 mg/kg, HS100: *Hibiscus sabdariffa* 100 mg/kg, HS200: *Hibiscus sabdariffa* 200 mg/kg, HS400: *Hibiscus sabdariffa* 400 mg/kg.

Ethanol administration decreased the brain [F (6, 28) = 82.4; P < 0.0001] and hepatic [F (6, 28) = 13.1; P < 0.0001] activities of catalase (Fig. 7A and B). *H. sabdariffa* aqueous calyxes extract at 100, 200, or 400 mg/kg significantly increased catalase activity, respectively to 0.07 ± 0.001, 0.05 ± 0.006, or 0.077 ± 0.006 nmol/g of tissue in the brain (compared to 0.003 ± 0.004 nmol/g tissue in the negative control group) (Fig. 7A). At 400 mg/kg, the extract significantly increased the hepatic catalase activity to 0.128 ± 0.007 nmol/g tissue against 0.068 ± 0.001 nmol/g tissue in the negative control group (Fig. 7B).

Diazepam induced an increase (P ≤ 0.01) in the hepatic and cerebral activity of catalase to 0.09 ± 0.001 and 0.115 ± 0.005 nmol/g tissue (Fig. 7A and B).

### 3.10 Effect of *H. sabdariffa* calyxes on serum evaluations (ALT, AST, creatinine, and uric acid levels) of mice undergoing alcohol withdrawal

Ethanol withdrawal in mice led to a significant increase in serum ALT [F (6, 28) = 80.9; P < 0.0001], AST [F (6, 28) = 56.2; P < 0.001], creatinine [F (6, 28) = 65.66; P < 0.001] and uric acid [F (6, 28) = 108; P < 0.001] levels compare to other groups. *H. sabdariffa* calyxes at 100, 200 or 400 mg/kg significantly (P ≤ 0.001) decreased the serum ALT respectively to 2.49 ± 0.128, 3.38 ± 0.17 or 3.45 ± 0.157 U/L compared to 7.12 ± 0.22 U/L in the negative control group; the respective doses also significantly (P ≤ 0.001) decreased the serum AST levels respectively to 3.22 ± 0.244, 2.90 ± 0.113, or 2.83 ± 0.188 U/L compared to7.19 ± 0.23 U/L in the negative control mice; and the serum creatinine levels (P ≤ 0.001) to 0.76 ± 0.006, 0.25 ± 0.008, or 0.30 ± 0.03 mg/dL as compared to 5.54 ± 0.034 mg/dL in the negative control group (Table 4).

**Table 4:** Effects of *H. sabdariffa* calyx on AST, ALT, creatinine, and uric acid levels.

Treatments (mg/kg)	ALT (U/L)	AST (U/L)	Creatinine (mg/dL)	Uric acid (mg/dL)
Normal control	2.49 ± 0.128	3.69 ± 0.435	0.58 ± 0.16	2.29 ± 0.21
Negative control	7.12 ± 0.22^###^	7.19 ± 0.23^###^	5.54 ± 0.034^###^	6.51 ± 0.07^###^
HS50	6.13 ± 0.16	6.58 ± 0.244	2.23 ± 0.0172∗∗∗	5.35 ± 0.26
HS100	2.49 ± 0.128∗∗∗	3.22 ± 0.244∗∗∗	0.76 ± 0.006∗∗∗	2.94 ± 0.08∗∗∗
HS200	3.38 ± 0.173∗∗∗	2.90 ± 0.113∗∗∗	0.25 ± 0.008∗∗∗	2.28 ± 0.11∗∗∗
HS400	3.46 ± 0.157∗∗∗	2.83 ± 0.188∗∗∗	0.30 ± 0.03∗∗∗	3.06 ± 0.03∗∗∗
Positive control	3.92 ± 0.28∗∗∗	2.57 ± 0.22∗∗∗	0.76 ± 0.020∗∗∗	3.80 ± 0.07∗∗∗

Values are the mean ± SEM. n = 5; ^###^P < 0.001vs Normal control ∗∗∗P ≤ 0.001; vs Negative control; HS50: *Hibiscus sabdariffa* 50 mg/kg; HS100: *Hibiscus sabdariffa* 100 mg/kg; HS200: *Hibiscus sabdariffa* 200 mg/kg; HS400: *Hibiscus sabdariffa* 400 mg/kg.

Aqueous extract of *H. sabdariffa* calyxes also decreased uric acid levels, from 6.51 ± 0.07 mg/dL in the negative control to 2.94 ± 0.08 or 2.28 ± 0.11 mg/dL in animals receiving the extract, respectively, at 200 or 400 mg/kg (Table 4).

Similarly, diazepam decreased the serum ALT, AST, creatinine, and uric acid levels to 23.92 ± 0.28 U/L, 2.56 ± 0.49 U/L, 0.76 ± 0.41 mg/dL, and 3.80 ± 0.07 mg/dL, respectively (Table 4).

## 4 Discussion

This study aimed to test the efficacy of *H. sabdariffa* aqueous extract against alcohol withdrawal anxiety in mice. AWS is a cluster of clinical symptoms that follow an abrupt interruption of ethanol drinking [16,44]. Chronic ethanol intake and then withdrawal lead to epigenetic modifications in the brain, which are critical aspects of sustaining ethanol addiction [45]. Anxiety is a behavioural disorder common among AWS patients [46]. Anxiety in rodents can be assessed using the EPM, the HB, and the OF tests [47].

The EPM is being intensively used to screen anxiolytic compounds. Drugs and plants that reduce anxiety increase the investigation of the EPM open branches [48]. The results obtained showed that treatment with *H. sabdariffa* extract increased open arm activities in the EPM of mice undergoing alcohol withdrawal, suggesting that anxiety induced by alcohol withdrawal was reversed. The results concord with those of Begum and Younus (2018), who found that *Hibiscus rosa* sinensis increases the exploration of the open arm in the EPM mice [48]. In addition, the *H. sabdariffa* extract administration reduced the exploration of the EPM closed arms. Based on the evidence that a reduction of activities in the closed arms reflects a reduction of anxiety [49], and that increases in open arms exploration reflect a reduction of anxiety in the EPM [47]. It could be suggested that the aqueous extract of calyxes of *H. sabdariffa* may exhibit anxiolytic activities against ethanol withdrawal.

The OFT assessed mice's exploration, locomotion, and emotional behaviour [50]. *H. sabdariffa* gavage increased the centre time of mice subjected to alcohol withdrawal in the OFT. This effect was identical to the effect of diazepam, a reference anxiolytic drug [51]. There was also a reduction in the faecal boli of animals treated with diazepam and *H. sabdariffa*. These results follow the same trends as those obtained by Moto and collaborators in 2018, who found a significant decrease in faecal boli in mice that received *Cissus quadrangularis* extract, revealing the potential anxiolytic activity [36].

The HBT is a behavioural test that evaluates fear in laboratory animals, regarding head dipping activities, which decreases in anxious animals [52]. Treating mice with *H. sabdariffa* significantly increases the number of head dips, suggesting decreased anxiety [38]. The results of the three above behavioural examinations point out the ameliorative properties of *H. sabdariffa* against ethanol withdrawal anxiety. These results could be attributed to the phytochemical richness of *H. sabdariffa* calyxes. The results of the phytochemical screening revealed that the Roselle calyxes contained flavonoids and polyphenols. These aforementioned compounds have been shown to exhibit anxiolytic activities via the modulation of the GABAergic and antioxidant pathways [53].

It is well stated in the literature that alcohol withdrawal produces oxidative stress via the glutamate excitatory neurotransmitter pathway [54,55]. Reactive oxygen species induced by alcohol withdrawal promote lipid peroxidation [55]. MDA is a biological indicator of lipid peroxidation. The reduction of this peroxidation in the liver and brain in mice undergoing alcohol withdrawal and treated with *H. sabdariffa* indicates that the aqueous extract of roselle calyxes has antioxidant power. Our results align with those of Janson and collaborators in 2021, who reported that *H. sabdariffa* calyx extract exhibits antioxidant activities [56].

NO content is also an indicator of the oxidative state of a biological tissue. In this study, alcohol termination significantly increased the NO in the brains and livers of mice in the negative control group. Administration of *H. sabdariffa* reduced NO levels in both organs, implicating a reduction of oxidative stress. Reduced glutathione is an enzyme that protects cells against oxidative damage. Its role relies mainly on increasing the activities of some free radical scavengers and the antioxidant vitamins (C and E) [57]. The antioxidant activity is conferred to the aqueous extract of *H. sabdariffa* calyxes by its potential to increase GSH levels in the liver and the brain of mice subjected to ethanol withdrawal; this increase in GSH activity is an indication of the significant antioxidant activity of a compound [58].

SOD plays a significant role in the prevention of tissue oxidation. SOD supplementation could be an effective preventive strategy to slow down the overproduction of free radicals during alcohol withdrawal [59]. In this work, results showed that the antioxidant activity is conferred to *H. Sabdariffa* by its capacity to increase SOD levels in the liver and brain of mice undergoing alcohol withdrawal at all different doses. Therefore, the extract of the calyxes of *H. sabdariffa* could have hepatoprotective and neuroprotective properties against oxidative stress induced by alcohol withdrawal in mice. The present findings corroborate those of Janson in 2021, who showed that Roselle calyx extract is protective against oxidative damage in obese rats fed with a fatty diet [56].

Serum assessment results revealed that alcohol administration raised ALT and AST. This aligns with previous reports, indicating an elevation of ALT and AST in ethanol intoxication [60,61]. The administration of *H. sabdariffa* calyx reduced serum ALT and AST, suggesting an ameliorative function of the liver in ethanol-treated mice. Creatinine and Uric acid levels increased in the serum of the negative control undergoing alcohol withdrawal. This increase in creatinine and uric acid is a sign of kidney damage [62]. Creatinine and uric acid concentrations were reduced in the groups treated with Roselle extract. These results imply that Roselle inhibited the nephrotoxic effect of ethanol. These results correlate with those reported by Swaroopa in 2013, who reported the protective effect of ginger administration in rats undergoing renal impairment induced by ethanol [63].

Phytochemical study revealed that *H. sabdariffa* calyxes contained alkaloids, polyphenols, flavonoids, and tannins. These phytochemicals are well-known for their antioxidant activities [53]. Furthermore, *H. sabdariffa* calyx has been reported to be rich in anthocyanin, which is a powerful antioxidant [56]. Thus, these bioactive compounds could have played an antioxidant, hepato, and/or neuroprotective role in alcohol withdrawal-induced anxiety and associated oxidative stress.

## 5 Conclusion

At this stage of our investigation, we can undoubtedly confirm that our results point to new findings about the potential remarkable anxiolytic activity of aqueous extract of *H. sabdariffa* calyxes against alcohol withdrawal-induced anxiety. These aforementioned activities are probably mediated through antioxidant mechanisms. Consequently, the aqueous extract of *H sabdariffa* calyx might have prospective clinical applications in the management of anxiety and multiple organ damage induced by alcohol. Hence, for a better valorisation of this plant in the treatment of alcohol use disorders, more exploration of the mechanism of action of the plant extract, along with some robust additional behavioural analysis in rodents of the active constituent accountable for its biological action, is essential.

## Consent for participation

Non-applicable.

## Consent to publish

Non-applicable.

## Ethical statement

This research protocol follows the University of Buea Institutional Animal Care and Use Committee (UB-IACUC) guidelines. The protocol approval number is UB-IACUC N֩08/2021.

## Funding

This research received laboratory reagents and equipment from the International Foundation for Science (IFS) [grant numbers: I-1-F-6443-1].

## CRediT authorship contribution statement

**Nadège Emégam Kouémou:** Conceptualization, Data curation, Formal analysis, Funding acquisition, Methodology, Writing – original draft, Writing – review & editing. **Louis Aimé Sepi:** Formal analysis, Writing – original draft. **Mireille Sylviane Nguepi Dongmo:** Investigation, Methodology, Writing – review & editing. **Ndzweng Linda Tamanji:** Investigation, Methodology, Writing – review & editing. **Franklin Savo Mbeboh:** Investigation, Methodology, Writing – review & editing. **Stephanie Jacqueline Kameni Ndjapdounke:** Investigation, Writing – review & editing. **Paul Aimé Noubissi:** Writing – review & editing. **Bernard Tiencheu:** Investigation, Methodology, Writing – review & editing. **Elisabeth Ngo Bum:** Supervision.

## Declaration of competing interest

The authors of this manuscript have no conflict of interest regarding the publication of this work.

## Data Availability

The data that support the findings of this study are available from the corresponding author upon reasonable request.
